# Excess Zinc Supply Reduces Cadmium Uptake and Mitigates Cadmium Toxicity Effects on Chloroplast Structure, Oxidative Stress, and Photosystem II Photochemical Efficiency in *Salvia sclarea* Plants

**DOI:** 10.3390/toxics10010036

**Published:** 2022-01-12

**Authors:** Ilektra Sperdouli, Ioannis-Dimosthenis S. Adamakis, Anelia Dobrikova, Emilia Apostolova, Anetta Hanć, Michael Moustakas

**Affiliations:** 1 Institute of Plant Breeding and Genetic Resources, Hellenic Agricultural Organization–Demeter, Thermi, 57001 Thessaloniki, Greece; 2 Section of Botany, Department of Biology, National and Kapodistrian University of Athens, 15784 Athens, Greece; iadamaki@biol.uoa.gr; 3 Institute of Biophysics and Biomedical Engineering, Bulgarian Academy of Sciences, 1113 Sofia, Bulgaria; aneli@bio21.bas.bg (A.D.); emya@bio21.bas.bg (E.A.); 4 Department of Trace Analysis, Faculty of Chemistry, Adam Mickiewicz University, 61614 Poznań, Poland; anettak@amu.edu.pl; 5 Department of Botany, Aristotle University of Thessaloniki, 54124 Thessaloniki, Greece

**Keywords:** phytotoxicity, clary sage, chlorophyll fluorescence, photosynthesis, reactive oxygen species, hydroponic culture, redox state, quinone A (Q*_A_*), non photochemical quenching, hydrogen peroxide

## Abstract

*Salvia sclarea* L. is a Cd^2+^ tolerant medicinal herb with antifungal and antimicrobial properties cultivated for its pharmacological properties. However, accumulation of high Cd^2+^ content in its tissues increases the adverse health effects of Cd^2+^ in humans. Therefore, there is a serious demand to lower human Cd^2+^ intake. The purpose of our study was to evaluate the mitigative role of excess Zn^2+^ supply to Cd^2+^ uptake/translocation and toxicity in clary sage. Salvia plants were treated with excess Cd^2+^ (100 μM CdSO_4_) alone, and in combination with Zn^2+^ (900 μM ZnSO_4_), in modified Hoagland nutrient solution. The results demonstrate that *S. sclarea* plants exposed to Cd^2+^ toxicity accumulated a significant amount of Cd^2+^ in their tissues, with higher concentrations in roots than in leaves. Cadmium exposure enhanced total Zn^2+^ uptake but also decreased its translocation to leaves. The accumulated Cd^2+^ led to a substantial decrease in photosystem II (PSII) photochemistry and disrupted the chloroplast ultrastructure, which coincided with an increased lipid peroxidation. Zinc application decreased Cd^2+^ uptake and translocation to leaves, while it mitigated oxidative stress, restoring chloroplast ultrastructure. Excess Zn^2+^ ameliorated the adverse effects of Cd^2+^ on PSII photochemistry, increasing the fraction of energy used for photochemistry (Φ*_PSII_*) and restoring PSII redox state and maximum PSII efficiency (*Fv*/*Fm*), while decreasing excess excitation energy at PSII (EXC). We conclude that excess Zn^2+^ application eliminated the adverse effects of Cd^2+^ toxicity, reducing Cd^2+^ uptake and translocation and restoring chloroplast ultrastructure and PSII photochemical efficiency. Thus, excess Zn^2+^ application can be used as an important method for low Cd^2+^-accumulating crops, limiting Cd^2+^ entry into the food chain.

## 1. Introduction

Increased industrial and agricultural human activities, such as mining and smelting, electroplating, wastewater irrigation, and chemical fertilizers, have resulted in high environmental content of cadmium (Cd^2+^) [1,2,3]. It is now well recognized that Cd^2+^, a non-essential element for plants that is not biodegradable in the soil, accumulates in the environment and subsequently becomes toxic to all living organisms [4,5,6,7,8].

Zinc (Zn^2+^) belongs to a group of eight essential micronutrients that are required for normal plant growth, development, and defense [9,10]. Zinc is involved in various metabolic processes, playing catalytic, regulatory, and structural roles with several crucial functions in the cell [9,10,11,12,13,14,15,16]. It performs a fundamental role in anti-oxidative defense and retains the membranous structure of various cell organelles [14,17]. Zn^2+^ deficiency or Zn^2+^ at high concentration in the soil, which occurs in various habitats, ranging from deficient to toxic levels can cause inhibition of numerous plant metabolic processes, reduce plant growth and photosynthesis, and decrease chlorophyll content in the leaf, provoking chlorosis and leaf necrosis [9,11,12,13,14]. The zinc homeostasis mechanism is not global within plants since most plants contain between 30 and 100 μg Zn^2+^ g^−1^ dry weight (DW), but some other species are accumulating more than 10,000 μg Zn^2+^ g^−1^ DW without showing symptoms of toxicity [18], despite the fact that concentrations above 300 μg Zn^2+^ g^−1^ DW are considered toxic to plants [1,9]. 

Accumulative concentration of Cd^2+^ in soil is highly alarming due to risk of its entrance into food chain, while foliar Cd^2+^ concentrations above 100 μg g^−1^ DW (0.01%) are considered exceptional and a threshold value for Cd-hyperaccumulation [1,19]. Cadmium availability in soils depends upon a large number of aspects such as clay minerals, organic matter, soil pH, cation exchange capacity (CEC), type of fertilizers, and soil Cd^2+^ content [20,21,22]. Zinc, owing to its chemical similarity with Cd^2+^, might act as a competitive ion for the binding sites both in the soil and root surfaces for uptake, and/or might interact with Cd^2+^ within the transport system of plants [22,23]. It is regarded as the principal micronutrient to ameliorate the toxic effect of Cd^2+^ on plants and to limit its entry into food chain [24]. Foliar application of Zn^2+^ ameliorated the adverse effects of Cd^2+^ and decreased grain Cd^2+^ of wheat grown in Cd^2+^-contaminated soil [24,25]. In addition, it decreased oxidative stress and increased nutrients, chlorophyll content, and photosynthetic rates, in Cd^2+^-stressed wheat seedlings [22,24,25]. The addition of Zn^2+^ to Cd^2+^-containing soils or nutrient solutions has been concluded to be successful in reducing Cd^2+^ accumulation in crop plants [22,25,26], but since plant responses vary with genotypes and the dose and duration of the Zn^2+^ and Cd^2+^ exposure, it is suggested that more studies are necessary to explore the proper Zn^2+^/Cd^2+^ ratios required to reduce Cd^2+^ toxicity [22].

Clary sage (*Salvia sclarea* L.), a biennial or a perennial 20–130 cm high plant that belongs to the Lamiaceae family, is native to Southern Europe but is being cultivated worldwide in temperate and sub-tropical climates as an ornamental and essential oil-bearing plant [27]. It is used in the aromatic and food industries [28], especially in alcoholic beverages, pastries, gelatins, puddings, frozen desserts, and condiments [27] and for preventing food spoilage due to its antimicrobial properties [29]. Its essential oils present antifungal and cytotoxic activity [30,31]. Recent studies reported analgesic and antidiabetic as well as anti-inflammatory effects [27]. *Salvia sclarea* is also an economically important plant for phytoextraction and phytostabilization of Cd^2+^- and Zn^2+^-contaminated soils, with an increasing interest in cultivation because of this [2,14,27].

We have previously observed that *S. sclarea* plants show photosynthetic tolerance to Cd^2+^ toxicity by increasing the photoprotective mechanism of non-photochemical quenching (NPQ) and accelerating the cyclic electron transport around photosystem I (PSI) that protects the function of the photosynthetic apparatus under excess Cd^2+^ [2]. Nonetheless, after exposure of *S. sclarea* to Cd^2+^ for up to 5 days, despite significant levels of Cd^2+^ in leaves, an enhanced photosystem II (PSII) functionality was observed, with a higher fraction of absorbed light energy to be directed to photochemistry (Φ*_PSII_*) and no defects on chloroplast ultrastructure to be noticed [3]. However, in *S. sclarea* plants exposed to the same level of Cd^2+^, but for 8 days, an inhibition of PSII functionality was observed, with the photoprotective mechanism of NPQ found to be inefficient in keeping the same fraction of open PSII reaction centers (q*_p_*) compared with non-treated plants [3]. In contrast, when *Salvia sclarea* L. was exposed to excess Zn^2+^ (900 μM) for 8 days, a stimulation of PSI and PSII activity was accompanied by increased synthesis of antioxidants in the leaves that play an important role in Zn^2+^ detoxification and protection against oxidative stress [14]. 

In the present study we hypothesized that excess Zn^2+^ supply to Cd^2+^-containing nutrient solution would decrease Cd^2+^ uptake and oxidative stress and would thus ameliorate PSII performance of *S. sclarea* plants exposed to Cd^2+^ toxicity. We compared Cd^2+^ uptake and toxicity effects, with and without Zn^2+^ supplementation, in order to evaluate the application of excess Zn^2+^ supply as a possible method to limit Cd^2+^ entry into the food chain through the medicinal herb clary sage. 

## 2. Materials and Methods

### 2.1. Plant Material and Growth Conditions

Seeds of *Salvia sclarea* L. collected from the Rose Valley (Bulgaria) were used for the experiments. After germination in a growth room on soil, for about a month, the seedlings were transferred for about one more month to pots in continuously aerated modified Hoagland nutrient solution that was changed every 3 days and described in detail before [2,8]. The growth room conditions were 14/10 h day/night photoperiod, with 200 ± 20 μmol photons m^−2^ s^−1^ photon flux density and 24 ± 1/20 ± 1 °C day/night temperature. 

### 2.2. Cadmium and Cadmium plus Zinc Treatments

Forty-five two-month-old *S. sclarea* plants were divided into three groups, and each group was subjected to hydroponic culture for 8 more days either (i) with Hoagland nutrient solution alone, which served as control with 5 μM Zn^2+^, (ii) with Hoagland nutrient solution with 100 μM Cd^2+^ (supplied as 3CdSO_4_.8H_2_O) [2], and (iii) with Hoagland nutrient solution with 100 μM Cd^2+^ (supplied as 3CdSO_4_.8H_2_O) plus 900 μM Zn^2+^ (supplied as ZnSO_4_) [14]. All solutions were renewed every 2 days so that nutrient content, Cd^2+^, and Zn^2+^ supply remained constant. The duration and the concentration of Cd^2+^ and Zn^2+^ treatments were based on our previous experiments with Salvia plants exposed to Cd^2+^ and Zn^2^ [3,14]. When *S. sclarea* plants were exposed for up to 5 days to 100 μM Cd^2+^, despite significant levels of Cd^2+^ in the leaves, an enhanced PSII functionality was observed, but when they were exposed to the same level of Cd^2+^ for 8 days, an inhibition of PSII functionality was noticed [3]. On the other hand, exposure of *S. sclarea* plants for 8 days to 900 μM Zn^2+^ enhanced PSII functionality [14]. Thus, in the present study, we used 8 days of exposure to 100 μM Cd^2+^ plus 900 μM Zn^2+^ to reveal any mitigative effect of Zn^2+^ on Cd^2+^ toxicity. 

### 2.3. Cadmium and Zinc Determination by Inductively Coupled Plasma Mass Spectrometry (ICP-MS)

After 8 days, five plants per group (control, Cd^2+^-, and Cd^2+^ + Zn^2+^-treated *S. sclarea*) were harvested, separated in roots and aboveground tissues, washed in deionized water, and then dried at 65 °C to constant biomass. Dried milled and sieved samples were further proceeded and digested in a microwave-assisted digestion system as described previously [3]. Samples were analyzed by an inductively coupled plasma mass spectrometry (ICP-MS) model ELAN DRC II (PerkinElmer Sciex, Toronto, ON, Canada) [32]. ICP-MS operational conditions, instrumental settings, calibration solutions, data validation, and validation parameters were as described before [3]. Elemental analysis was performed for Cd^2+^ and Zn^2+^.

### 2.4. Chlorophyll Fluorescence Analysis

Chlorophyll fluorescence measurements were conducted on dark adapted leaves of five plants per group (control, Cd^2+^, and Cd^2+^ + Zn^2+^), using an *Imaging PAM M-Series* system (*Heinz Walz Instruments*, Effeltrich, Germany) as described in detail previously [33]. In each leaf, representative areas of interest (AOIs) were selected, and the chlorophyll fluorescence parameters F*o* (minimum chlorophyll *a* fluorescence in the dark-adapted leaf), F*m* (maximum chlorophyll *a* fluorescence in the dark-adapted leaf), Fo’ (minimum chlorophyll *a* fluorescence in the light-adapted leaf), F*m’* (maximum chlorophyll *a* fluorescence in the light-adapted leaf), and F*s* (steady-state photosynthesis at 900 μmol photons m^–2^ s^–1^ actinic light (AL) intensity) were measured [34]. The chlorophyll fluorescence parameters calculated by the Imaging Win V2.41a software (Heinz Walz GmbH, Effeltrich, Germany) from the above parameters were: *Fv*/*Fm* (maximum efficiency of PSII photochemistry); Φ*_PSII_* (the actual quantum yield of PSII photochemistry); Φ*_NPQ_* (the quantum yield of regulated non-photochemical energy loss in PSII); Φ*_NO_* (the quantum yield of non-regulated energy dissipated in PSII); the redox state of quinone A (Q*_A_*), an estimate of the fraction of open PSII reaction centers based on the “puddle” model for the photosynthetic unit (*qp* = [F*m*′ − F*s*]/[F*m*′ − F*o*′]); q*_L_* (the fraction of open PSII reaction centers that are connected by shared antenna, that is, the so-called “lake” model) [35]; and EXC, the relative excess energy at PSII, (EXC = [*Fv*/*Fm* − Φ*_PSII_* ]/[*Fv*/*Fm*]), according to Bilger et al. [36].

### 2.5. Determination of Oxidative Damage and Hydrogen Peroxide

The oxidative damage in *S. sclarea* leaves was estimated by the level of lipid peroxidation according to the method of Hodges et al. [37]. Leaf samples from five plants per group (control, Cd^2+^, and Cd^2+^ + Zn^2+^), after 8 days of treatment, were frozen in liquid nitrogen and stored at −80 °C before the evaluation of malondialdehyde (MDA) content by the reaction with 2-thiobarbituric acid (TBA), as described before [3]. Frozen leaf tissues were homogenized at 4 °C in 1% (*w*/*v*) trichloroacetic acid (TCA) and then centrifuged at 14,000× *g* for 20 min. Absorbance of the supernatant was read at 532 nm using Specord 210 Plus (Ed. 2010, Analytik Jena AG, Germany). Results are expressed as μmol MDA g^−1^ FW.

Hydrogen peroxide (H_2_O_2_) generation was estimated in leaves from the same *S. sclarea* plants used for MDA evaluation after extraction by homogenization with 50 mM K-phosphate buffer pH (6.5) and reaction with 0.1% TiCl_4_ in 20% H_2_SO_4_, as described before [3].

### 2.6. Chloroplast Ultrastructure Observations

Chloroplast ultrastructure alterations were observed in the same plants that were used for chlorophyll fluorescence measurements after 8 days of treatment. Leaf segments of 0.5 × 1 mm from control, Cd^2+^-, and Cd^2+^ + Zn^2+^-treated plants were fixed with 2% paraformaldehyde plus 4% glutaraldehyde in 0.05 M sodium cacodylate buffer at pH 7.0 as described previously [28]. Fixation took place at room temperature for 5 h and post-fixation for another 3 h and further treatments as described in detail before [3]. Ultrathin sections (80–90 nm) were stained with 2% uranyl acetate and 1% lead citrate and examined in a JEOL JEM 1011 transmission electron microscope equipped with a Gatan ES500W digital camera. Digital electron micrographs were obtained with the DigitalMicrograph 3.11.2 software (Digital Micrograph Gatan, Oxon, UK) [3].

### 2.7. Statistical Analysis

Statistical analysis was performed by one-way ANOVA analysis followed by post hoc comparisons using Dunn–Šidák correction. Means (± SD) were calculated from five independent biological replicates and were considered statistically different at a level of *p* < 0.05. Data analysis was performed with IBM SPSS Statistics for Windows version 28.0.

## 3. Results

### 3.1. Cadmium and Zinc Accumulation in Leaves and Roots of Salvia sclarea in Response to Cadmium Toxicity with and without Zinc Application

**Figure 1:**
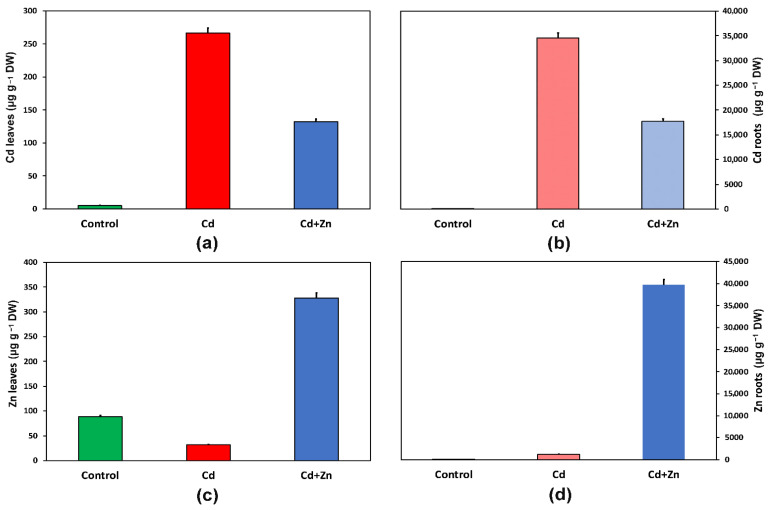
Changes in Cd^2+^ accumulation in leaves (**a**) and roots (**b**) and Zn^2+^ accumulation in leaves (**c**) and roots (**d**) in µg g^−1^ dry weight (DW) after eight days treatment of *Salvia sclarea* plants with control (nutrient solution), Cd^2+^ (nutrient solution +100 μM Cd^2+^), and Cd^2+^ + Zn^2+^ (nutrient solution +100 μM Cd^2+^ + 900 μM Zn^2+^). Error bars are standard deviations (*n* = 5).

Exposure of *S. sclarea* plants to 100 μM Cd^2+^ for 8 days, increased Cd^2+^ in leaves by 50-fold (*p* < 0.05) (Figure 1a) and of roots by 7000-fold (Figure 1b), as Cd^2+^ in the roots reached 34,503 ± 1035 µg g^−1^ DW vs. 266.3 ± 7.8 µg g^−1^ DW in the leaves, as also reported previously [2]. Addition of 900 μM Zn^2+^ to the nutrient solution with 100 μM Cd^2+^ resulted in almost 50% decreased Cd^2+^ content in leaves (131.9 ± 3.9 µg g^−1^ from 266.3 ± 7.8 µg g^−1^ DW) (Figure 1a) and roots (17,704 ± 531 µg g^−1^ from 34,503 ± 1035 µg g^−1^ DW) (Figure 1b).

Exposure to Cd^2+^ enhanced total Zn^2+^ uptake by 7.0-fold but decreased its translocation to the leaves by 36% (Figure 1c). Zinc content in roots after Cd^2+^ exposure reached 1228 ± 37 µg g^−1^ DW, from 89.3 ± 2.7 µg g^−1^ DW (Figure 1d), while in leaves from 88.1 ± 2.6 µg g^−1^ DW decreased to 31.8 ± 0.96 µg g^−1^ DW (Figure 1c). Zinc content in leaves, after Zn^2+^ supplementation with Cd^2+^, reached 328.1 ± 9.8 µg g^−1^ DW (10-fold increase) (Figure 1c), while in roots 39,711 ± 1191 DW µg g^−1^ dry weight (32-fold increase) (Figure 1d).

### 3.2. Changes in the Light Energy Utilization in Photosystem II in Response to Cadmium Toxicity with and without Zinc Application

We evaluated the light energy distribution pattern in PSII for photochemistry (Φ*_PSΙΙ_*) for regulated non-photochemical energy loss—that is, for photoprotective heat dissipation (Φ*_NPQ_*)—and for non-regulated energy loss in PSII (Φ*_NO_*). The addition of these three fractions is unity [35,38,39,40,41].

**Figure 2:**
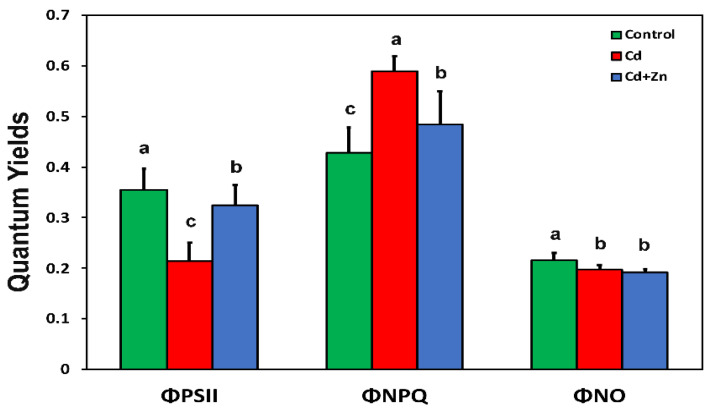
Changes in photosystem II quantum yields of *Salvia sclarea* control (nutrient solution), Cd^2+^ (nutrient solution +100 μM Cd^2+^), and Cd^2+^ + Zn^2+^ (nutrient solution +100 μM Cd^2+^ + 900 μM Zn^2+^). Effective quantum yield of photochemistry (Φ*_PSΙΙ_*), regulated non-photochemical energy loss (Φ*_NPQ_*), and non-regulated energy loss in PSII (Φ*_NO_*) at 900 μmol photons m^−2^ s^−1^ actinic light (AL) intensity. Error bars are standard deviations (*n* = 5). Columns in each chlorophyll fluorescence parameter with different lowercase letters are statistically different (*p* < 0.05).

In Cd^2+^-treated *S. sclarea* plants, the light energy used for photochemistry (Φ*_PSII_*) decreased, while the photoprotective energy dissipation as heat (Φ*_NPQ_*) increased compared with control *Salvia* plants, with a concomitant decrease in the fraction of non-regulated energy lost (Φ*_NO_*) (Figure 2).

The application of 900 μM Zn^2+^ in the nutrient solution containing 100 μM Cd^2+^ increased the fraction of energy used for photochemistry (Φ*_PSII_*) and decreased the fraction of heat dissipation (Φ*_NPQ_*), while there was no difference in the fraction of non-regulated loss (Φ*_NO_*) compared with Cd^2+^ alone (Figure 2).

### 3.3. Changes in the Maximum Efficiency of Photosystem II and the Redox State of Quinone A (Q_A_) to Cadmium Toxicity with and without Zinc Application

**Figure 3:**
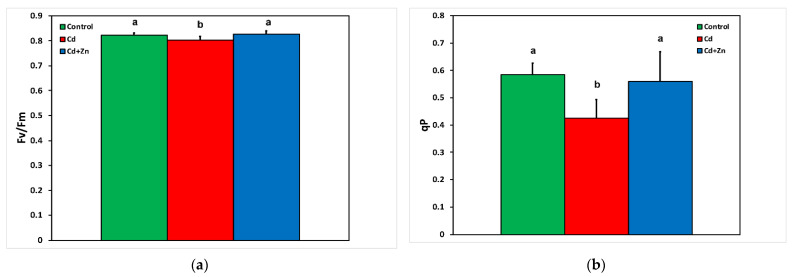
Changes in the maximum efficiency of photosystem II (*Fv*/*Fm*) (**a**), and the redox state of quinone A (Q*_A_*), an estimate of the fraction of open PSII reaction centers estimated at 900 μmol photons m^–2^ s^–1^ actinic light (AL) intensity (*qp*) (**b**), of *Salvia sclarea* control (nutrient solution), Cd^2+^ (nutrient solution +100 μM Cd^2+^), and Cd^2+^ + Zn^2+^ (nutrient solution +100 μM Cd^2+^ + 900 μM Zn^2+^). Error bars are standard deviations (*n* = 5). Columns with different lowercase letters are statistically different (*p* < 0.05).

In Cd^2+^-treated *S. sclarea* plants, both the maximum efficiency of PSII (*Fv*/*Fm*) (Figure 3a) and the redox state of quinone A (Q*_A_*), an estimate of the fraction of open PSII reaction centers (*qp*) (Figure 3b), decreased, while the application of Zn^2+^ in the nutrient solution increased both of them to the level of control *Salvia* plants.

### 3.4. Changes in the Redox State of Plastoquinone Pool Based on the Lake Model and the Excess Excitation Energy in Response to Cadmium Toxicity with and without Zinc Application

**Figure 4:**
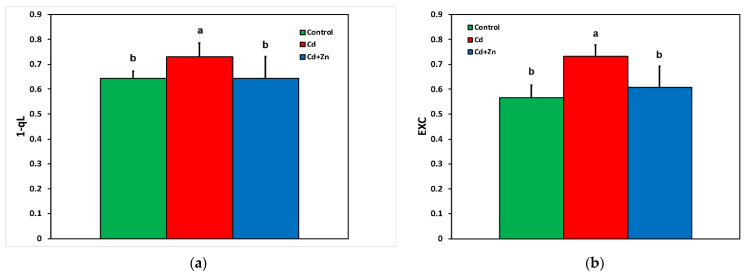
Changes in the redox state of plastoquinone pool based on the lake model (1 − q*_L_*) (**a**), and the excess excitation energy (EXC) (**b**), estimated at 900 μmol photons m^−2^ s^−1^ actinic light (AL) intensity, of *Salvia sclarea* control (nutrient solution), Cd^2+^ (nutrient solution +100 μM Cd^2+^), and Cd^2+^ + Zn^2+^ (nutrient solution +100 μM Cd^2+^ + 900 μM Zn^2+^). Error bars are standard deviations (*n* = 5). Columns with different lowercase letters are statistically different (*p* < 0.05).

The redox state of plastoquinone pool based on the lake model (1 − q*_L_*) (Figure 4a) and the excess excitation energy (EXC) (Figure 4b), both measured at 900 μmol photons m^−2^ s^−1^ actinic light (AL) intensity, increased in Cd^2+^-treated *S. sclarea* plants, but both decreased to the level of control *Salvia* plants with the application of Zn^2+^ in the nutrient solution.

### 3.5. Changes in the Level of Lipid Peroxidation and Hydrogen Peroxide (H_2_O_2_) Generation in Response to Cadmium Toxicity with and without Zinc Application

**Figure 5:**
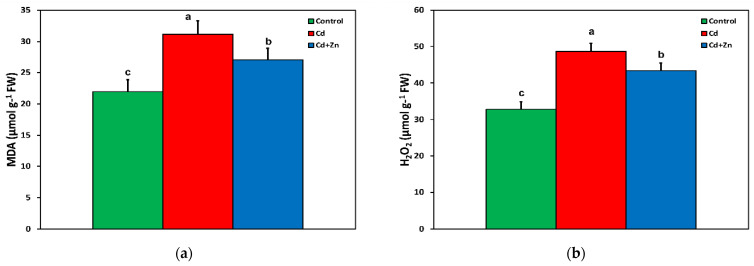
Changes in the level of lipid peroxidation, assessed by the accumulation of malondialdehyde (MDA) (**a**), and the generation of hydrogen peroxide (H_2_O_2_) (**b**), in the leaves of *Salvia sclarea* control (nutrient solution), Cd^2+^ (nutrient solution +100 μM Cd^2+^), and Cd^2+^ + Zn^2+^ (nutrient solution +100 μM Cd^2+^ + 900 μM Zn^2+^). Error bars are standard deviations (*n* = 5). Columns with different lowercase letters are statistically different (*p* < 0.05).

In Cd^2+^-treated *S. sclarea* plants, the increased accumulation of malondialdehyde (MDA) (Figure 5a) coincided with the increased generation of hydrogen peroxide (H_2_O_2_) (Figure 5b). However, both decreased with the application of Zn^2+^ in the nutrient solution but remained higher than the level of control *Salvia* plants (Figure 5a,b).

### 3.6. Changes in Chloroplast Ultrastructure to Cadmium Toxicity with and without Zinc Application

**Figure 6:**
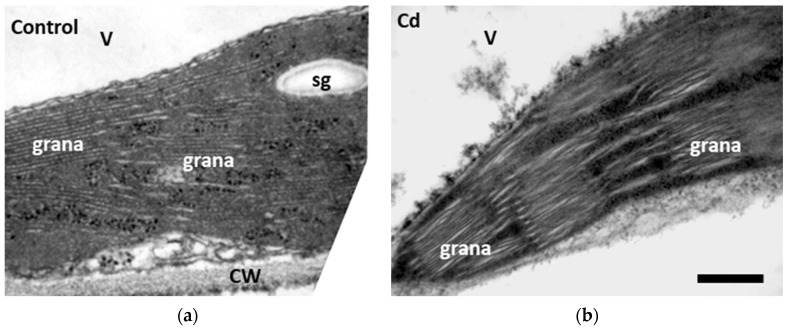
Transmission electron microscopy (TEM) images of chloroplasts from control (nutrient solution) (**a**) and 8-day Cd^2+^-treated (nutrient solution + 100 μΜ Cd^2+^) (**b**) *Salvia sclarea* leaves. Upon Cd treatment, chloroplasts appeared electronically dense and with swollen thylakoids, while being devoid of starch grains when compared with control. **CW**: cell wall; **sg**: starch grain; **V**: vacuole. Scale bar: 200 nm.

**Figure 7:**
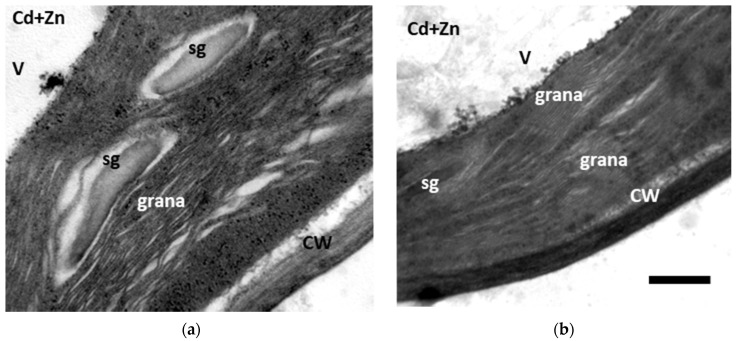
Transmission electron microscopy (TEM) images of chloroplasts from 8-day Cd^2+^+Zn^2+^-treated (nutrient solution + 100 μΜ Cd^2+^+ 900 μΜ Zn^2+^) *Salvia sclarea* leaves. Zn addition to the nutrient solution mitigated Cd effects, with starch grains being present (**a**), and the thylakoid membranes being less dilated (**b**). **CW**: cell wall; **sg**: starch grain; **V**: vacuole. Scale bar: 200 nm.

Chloroplast of control plants had a typical internal structure, with well-organized grana thylakoids and stroma membranes and with high electron opacity of the stroma (Figure 6a). After 8 consecutive days of Cd^2+^ application, effects on chloroplasts ultrastructure involved thylakoid disorganization, dilated thylakoid membranes, and the absence of starch grains (Figure 6b). Zn^2+^ supplementation seemed to alleviate Cd effects to the chloroplasts, with starch grains being present (Figure 7a) and the thylakoid membranes being less dilated (Figure 7b).

## 4. Discussion

*Salvia sclarea* is recognized as a medicinal herb due to its valuable pharmacological properties and numerous health benefits, being also an economically important plant for phytoextraction and phytostabilization of Cd^2+^- and Zn^2+^-contaminated soils, with a subsequent increased interest in its cultivation [2,14,27]. Cadmium is greatly injurious to plant growth and almost all human individuals are exposed to Cd^2+^, mostly through plant-derived food that has reached the threshold for adverse health effects, and therefore, there is a serious demand to lower human Cd^2+^ intake by development of low Cd^2+^-accumulating crops [42,43]. In addition to food consumption, medicinal plants, and processed foods, Cd^2+^ is naturally stored at high concentrations in cigarettes [42,43,44]. Cadmium accumulation in leaf tissues damages the photosynthetic machinery, reducing photosynthetic activity [45] and increasing reactive oxygen species (ROS) accumulation, resulting in oxidative stress, programmed cell death, and necrosis [43,46,47,48,49].

Reactive oxygen species, such as singlet oxygen (^1^O_2_), superoxide anion radical (O_2_**^•^**^−^), and hydrogen peroxide (H_2_O_2_), produced in plant cells, mainly in the electron transport chain of chloroplasts, are kept in a homeostasis by the antioxidative enzymatic and non-enzymatic systems [50,51,52,53,54]. However, under miscellaneous environmental stress conditions, the absorbed light energy surpasses what it can be used for in photochemistry, developing an increased ROS production that can cause cellular damage by oxidation of DNA, proteins, and lipids, resulting in oxidative stress [34,55,56]. Oxidative stress that is usually assessed by MDA content, a marker of lipid peroxidation [34,57,58], was found to increase under Cd^2+^ treatment (Figure 5a). However, Zn^2+^ supplementation to Cd^2+^-containing nutrient solution decreased lipid peroxidation, which remained higher compared with control values (Figure 5a), suggesting an increased ROS production. Under optimal growth conditions, a homeostasis of ROS is maintained [59,60], while an alteration in this homeostasis activates defense responses [3,59,61,62], but an elevated ROS level is harmful to plants [59,61]. However, ROS are considered to be essential signaling molecules that adjust plant development and the defense responses to various biotic and abiotic stresses [50,60,63,64]. A proper response to a stressor depends primarily on how plants identify the stress signal and respond to initiate a series of signaling cascades for initiation of acclimation mechanisms [65]. Cadmium-induced ROS, such as O_2_**^•^**^−^ and H_2_O_2_, is attributed to the phytotoxic effect of Cd^2+^, but ROS can also function as signal molecules in the induction of defense genes against Cd^2+^ toxicity [46]. The modulation of signal transduction pathways can also protect plants against Cd^2+^-induced cell death [47,66].

Several methods have been developed to remove or stabilize soil heavy metals and to reduce their accumulation in crops [67,68,69]. Mechanisms that interfere with Cd^2+^ uptake and accumulation have been shown to reduce Cd^2+^ toxicity [22,24,25,66,70,71]. Zinc, melatonin, and salicylic acid application are alleviating Cd^2+^-induced toxicity by inhibition of ROS overproduction [22,72,73]. Application of Zn^2+^ to a nutrient solution containing Cd^2+^ increased the fraction of energy used for photochemistry (Φ*_PSII_*) compared with Cd^2+^ alone, but Φ*_PSII_* remained lower compared with control *Salvia* plants (Figure 3). However, the fraction of non-regulated loss (Φ*_NO_*) decreased in both Cd^2+^ alone and Cd^2+^ + Zn^2+^-treated plants, compared with control *Salvia* plants (Figure 2). Overexcitation of PSII increases the probability of the formation of the triplet chlorophyll state (^3^Chl*) from the singlet excited state (^1^Chl*) through intersystem crossing, producing single oxygen (^1^O_2_) [38,74,75,76,77,78,79,80]. This non-regulated energy loss in PSII is reflected by Φ*_NO_* [38,75,76,78,80]. Thus, Cd^2+^ alone, and Cd^2+^ + Zn^2+^-treated plants displayed less ROS production, as ^1^O_2_ (Figure 2), but increased total ROS due to increased H_2_O_2_ generation, compared with control plants (Figure 5b).

In Cd^2+^-treated plants, hydrogen peroxide (H_2_O_2_) generation increased by 48% compared with control leaves (Figure 5b), while the accumulation of MDA, indicating the degree of oxidative stress causing lipid peroxidation, increased by 42% (Figure 5a). The lower increase in the degree of oxidative stress may be attributed to the decrease in ROS production as ^1^O_2_, assessed by the decreased fraction of non-regulated loss (Φ*_NO_*) (Figure 2). Reactive oxygen species are formed by energy transfer (^1^O_2_) and electron transport (H_2_O_2_) simultaneously, and it seems that their action interferes with their signaling pathways, sometimes to antagonize each other [3]. It has been often shown that hydrogen peroxide diffuses through leaf veins to act as a long-distance molecule activating stress defense responses under biotic and abiotic stresses in plants [52,59,60,76,78]. The increased H_2_O_2_ generation in Cd^2+^ + Zn^2+^-treated plants (Figure 5b) seems to have triggered a defense response that lowered Cd^2+^ toxicity effects on PSII photochemistry by increasing the fraction of energy used for photochemistry (Φ*_PSII_*) (Figure 2), restoring the PSII redox state (Figure 3b) and the maximum PSII efficiency (*Fv*/*Fm*) (Figure 3a), suggesting beneficial effects of ROS production [59]. Under Cd^2+^ treatment alone, when the PQ pool (monitored by the 1 − q*_L_*) was highly reduced (Figure 4a), an excess excitation energy (EXC) (Figure 4b) was observed in Cd^2+^-treated Salvia plants. High excess excitation energy, and therefore an imbalance between energy supply and demand, results in overproduction of ROS [3,39,79,80], having as a consequence oxidative stress, which was detected by the increased MDA content, representing the degree of lipid peroxidation (Figure 5a).

A dose-related negative impact of Cd^2+^ that raises ROS concentration, inducing oxidative damage and inhibition of photosynthetic function, has been extensively reported [81,82,83,84,85,86,87,88]. However, stimulation of the photosynthetic rate by Cd^2+^ at low concentrations has also been described, with a concomitant altered ROS homeostasis [3,89,90]. Plants employ several enzymic and non-enzymatic antioxidative systems to remove the different types of ROS, thus diminishing possible cell damage [52,76,89,90]. The alteration of ROS homeostasis has been suggested to be the mechanism of Cd^2+^-induced hormetic response of photosynthesis in medicinal herbs [3,90]. An enhanced PSII photochemistry, indicating an “over-compensation” response to Cd^2+^ exposure was corelated with Cd^2+^ disruption of ROS homeostasis [3], validating the declaration of Carvalho et al. [91] that Cd^2+^ can also be considered a beneficial element as well as a toxic one. However, in our experiment, in Cd^2+^-treated plants, the light energy used for photochemistry (Φ*_PSII_*) decreased (Figure 2), and at the same time, the accumulation of MDA, indicating the degree of oxidative stress, increased (Figure 5a). Insufficient supply of energy for photochemistry decreases photosynthesis efficiency, which is detrimental to plant growth [92]. Zinc application to the nutrient solution, together with Cd^2+^, resulted in a 10-fold increase in Zn^2+^ in the leaves, which increased the fraction of energy used for photochemistry, compared with Cd^2+^ alone (Figure 2), while it decreased oxidative stress by 17%, also compared with Cd^2+^ alone (Figure 5a). Zinc, as a component of antioxidant enzymes, is essential for scavenging H_2_O_2_ and O_2_**^•^**^−^ [93]. Zinc application reduces Cd^2+^ accumulation, which depends greatly on the crop cultivar, and increases leaf Zn^2+^ content and at the same time decreases MDA concentration [94,95,96,97,98]. Application of selenium, silicon, melatonin, and salicylic acid have been also found to alleviate Cd^2+^-induced toxicity by inhibition of ROS overproduction [72,73,99,100,101,102,103].

It is well supported that Cd^2+^ negatively influences chloroplast structure, with observed defects that include thylakoid membrane dismantling, increased presence of plastoglobuli, starch grain decrease, and plastid envelope rupture [104]. The above are also observed in hyperaccumulator or Cd^2+^-tolerant plants when a threshold of Cd^2+^ concentration and duration treatment is surpassed [3,105,106]. In Cd^2+^-treated plants, chloroplast structure is compromised, with Cd^2+^ causing thylakoid membrane disruption and starch grain loss (Figure 6b). Increased ROS production (Figure 5b) could lead to a noticeable increase in membrane damage, indicated by the elevated MDA levels (Figure 5a), and the subsequent changes in chloroplast ultrastructure could specify dysfunctions in metabolism, indicating defects of the photosynthetic parameters (Figure 2). The observed decrease in photosynthetic parameters can, in turn, be associated with reduced carbon fixation [107] and consequently a lower number of starch grains [106]. In Cd^2+^ + Zn^2+^-treated plants, Cd^2+^-related toxic effects are ameliorated, with starch grains being present and the thylakoid membranes being less affected (Figure 7a,b). Generally, it has been stated that exogenous Zn^2+^ application alleviates oxidative damage induced by Cd^2+^ application (Figure 5a) since Zn^2+^ improved the function of the antioxidant systems [108,109], a notion mirrored in the chloroplast structure as well (Figure 7a,b).

Exposure of *S. sclarea* plants to 100 μM Cd^2+^ for 8 days resulted in 266 µg g^−1^ DW Cd^2+^ concentration in the leaves (Figure 1a). Addition of Zn^2+^ resulted in 50% decreased Cd^2+^ content in leaves (132 µg g^−1^ DW) (Figure 1a), and thus, it can be regarded as the principal element for ameliorating the toxic effects of Cd^2+^ on plants and limiting its entry into the food chain [24]. The average daily intake of Cd^2+^ via food in European countries and North America is 15-25 μg, while in Japan it is commonly 40–50 μg, but it may be considerably higher in Cd^2^-polluted areas [110]. Zinc content in *S. sclarea* leaves, after Zn^2+^ supplementation with Cd^2+^, reached 0.328 mg g^−1^ DW (Figure 1c), while daily Zn^2+^ intake must not exceed 40 mg per day for adults [111]. Proper Zn^2+^ biofortification, however, requires specification of plant species and soil type [111,112].

Our data on chlorophyll *a* fluorescence analysis as well as previous data indicated that Cd^2+^ exposure results in a partial inactivation of PSII reaction centers and inhibition of PSII functionality, disturbing electron transport in the oxygen-evolving complex [6,7,8,113,114,115]. Chlorophyll *a* fluorescence analysis is a promising technique for quick detection of photosynthetic efficiency, permitting short- or long-term abiotic or biotic stress impact on the mechanisms of PSII functionality to be revealed [116,117,118], while the use of chlorophyll fluorescence imaging analysis permits the detection of spatiotemporal heterogeneity at the total leaf surface [119]. Additional studies will be performed to elucidate defense mechanisms, including antioxidants, PSI-dependent cyclic electronic transport, and kinetics of oxygen-evolving reactions, which will provide more detailed information on the role of excess Zn in the increased Cd tolerance of *Salvia sclarea*.

## 5. Conclusions

Exposure of the medicinal herb *Salvia sclarea* to 100 μM Cd^2+^ for 8 consecutive days enhanced total Zn^2+^ uptake but decreased its translocation to the leaves and resulted in an inhibition of PSII functionality, with the photoprotective mechanism of the dissipation of the excess energy as heat to be ineffective in keeping the redox state of quinone A (Q*_A_*) oxidized at the same level as non-treated plants. Application of Zn^2+^ effectively mitigated Cd^2+^-induced toxicity on *Salvia sclarea* by reducing Cd^2+^ uptake, together with its translocation to the leaves, while it mitigated oxidative stress that restored partially the chloroplast ultrastructure. Excess Zn^2+^ ameliorated PSII photochemistry by increasing the fraction of energy used for photochemistry (Φ*_PSII_*) and restoring the PSII redox state and maximum PSII efficiency (*Fv*/*Fm*), while it lowered the excess excitation energy at PSII (EXC). We conclude that excess Zn^2+^ reduced Cd^2+^ uptake and translocation and restored chloroplast ultrastructure and PSII photochemical efficiency. Our results show that Zn^2+^ application on clary sage, by decreasing Cd^2+^ uptake to half, can be regarded as a fundamental method to limit Cd^2+^ entry into the food chain.

## Data Availability

The data presented in this study are available in this article.
